# Disruption of SETD3‐mediated histidine‐73 methylation by the BWCFF‐associated β‐actin G74S mutation

**DOI:** 10.1002/1873-3468.70088

**Published:** 2025-06-09

**Authors:** Anja Marquardt, Marcus S. Münchhoff, Jacqueline Krohn, Philip M. Palarz, Manuel H. Taft, Johannes N. Greve, Nataliya Di Donato, Falk F. R. Buettner, Dietmar J. Manstein

**Affiliations:** ^1^ Institute for Biophysical Chemistry Fritz–Hartmann–Centre for Medical Research, Hannover Medical School Germany; ^2^ Division for Structural Biochemistry Hannover Medical School Germany; ^3^ Department of Human Genetics Hannover Medical School Germany; ^4^ Institute of Clinical Biochemistry Hannover Medical School Germany; ^5^ Proteomics, Institute of Theoretical Medicine, Faculty of Medicine University of Augsburg Germany

**Keywords:** actinopathy, Baraitser–Winter Cerebrofrontofacial Syndrome, histidine methylation, SETD3, β‐actin

## Abstract

Histidine‐73 methylation of β‐actin by SETD3 modulates ATPase activity, filament assembly, and protein interactions. The pathogenic G74S mutation in cytoskeletal β‐actin, associated with Baraitser–Winter cerebrofrontofacial syndrome (BWCFF), alters the adjacent phosphate sensor loop, disrupting SETD3‐mediated methylation. Molecular docking indicates that SETD3 undergoes structural rearrangements to accommodate the mutant β‐actin, leading to reduced catalytic efficiency. Enzymatic assays confirm slower turnover of mutant actin peptides, while mass spectrometry reveals decreased histidine‐73 methylation in both recombinant mutant β‐actin and patient‐derived fibroblasts. This perturbance of SETD3‐mediated methylation likely generates β‐actin pools with distinct methylation states, varying across cell types and developmental stages, thereby impairing cytoskeletal dynamics and contributing to BWCFF pathology.

**Impact statement**

This study reveals that the BWCFF‐linked G74S mutation in β‐actin disrupts SETD3‐mediated histidine‐73 methylation, impairing a critical post‐translational modification. It provides the first direct mechanistic link between a cytoskeletal actinopathy and altered methylation, highlighting potential targets for therapeutic intervention in β‐actin‐related developmental disorders.

## Graphical abstract

The β‐actin G74S mutation causes altered interaction of actin with SETD3, reducing histidine‐73 methylation efficiency and forming two distinct actin variants. The variable ratio of these variants across cell types and developmental stages contributes to tissue‐specific phenotypical changes. This imbalance may impair actin dynamics and mechanosensitive signaling, compromising cellular homeostasis and contributing to disease pathogenesis.

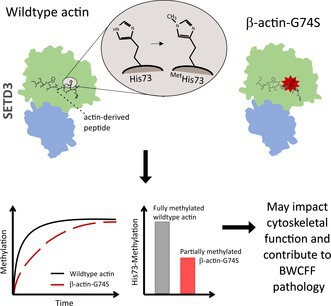

## Abbreviations

**ABP**, actin‐binding protein

**AF3**, AlphaFold 3

**AI**, artificial intelligence

**BWCFF**, Baraitser–Winter cerebrofrontofacial syndrome

**ESI**, electrospray ionization

**LSMT**, large subunit methyltransferase

**MS**, mass spectrometry

**NMA**, non‐muscular actinopathy

**PTM**, posttranslational modification

**SAH**, S‐adenosyl homocysteine

**SAM**, S‐adenosyl methionine

**SETD3**, SET domain‐containing 3

**vdW**, van der Waals

Cytoskeletal β‐ and γ‐actin, encoded by the *ACTB* and *ACTG1* genes, form the fundamental building blocks of the actin cytoskeleton in human cells [1]. These isoforms are crucial for determining cell shape and facilitating key processes such as migration, adhesion, division, and signal transduction. The dynamic remodeling of the actin cytoskeleton—driven by actin‐binding proteins (ABPs)—ensures precise control over actin filament assembly and disassembly, thereby enabling cellular plasticity and specialized cytoskeletal architectures [2, 3, 4, 5, 6]. The ATP‐dependent turnover of these actin networks, coupled with coordinated ABP interaction, is essential for processes such as directed migration and mechano–transduction.

**Fig. 1:**
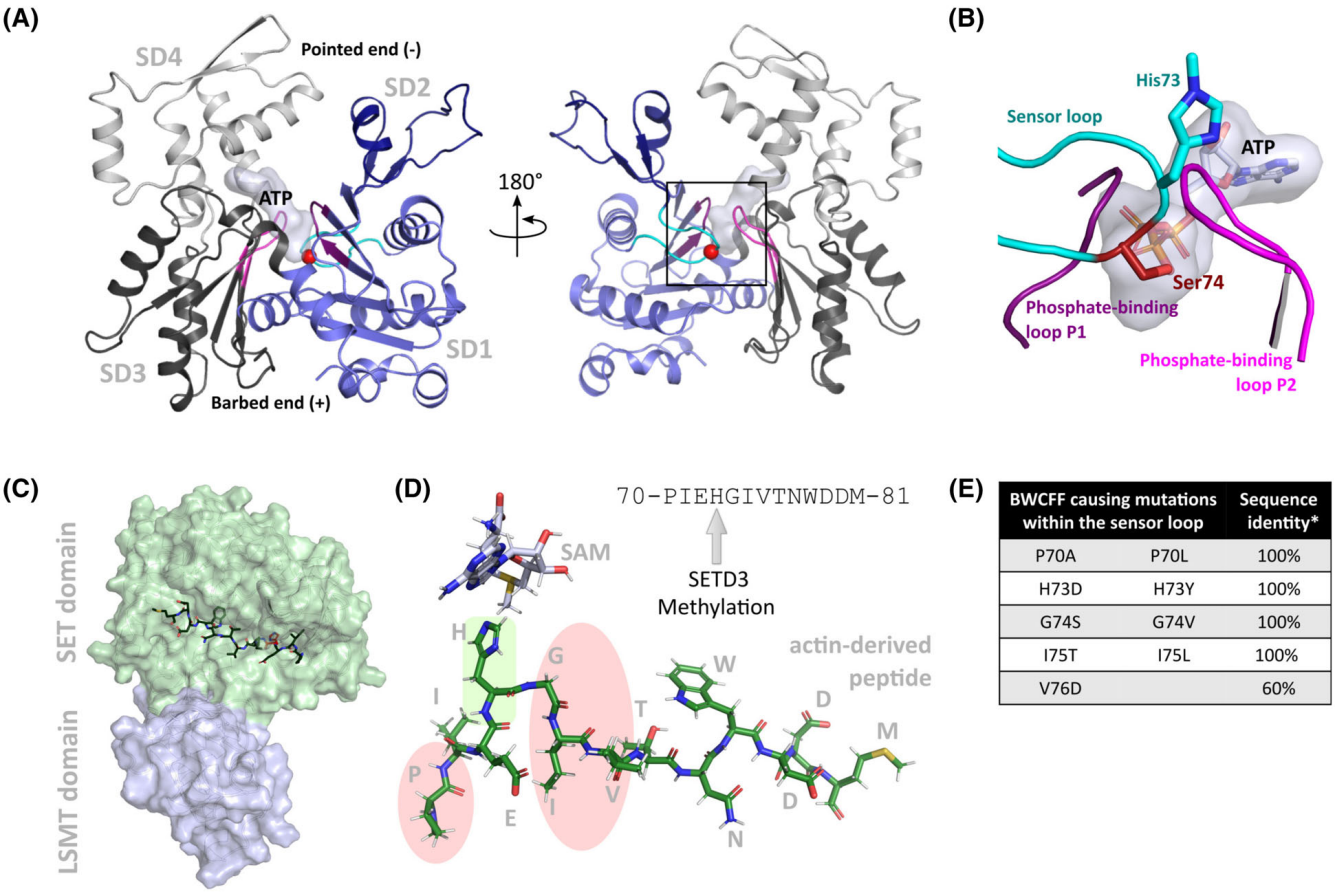
The actin sensor loop is a hotspot for BWCFF‐causing mutations. (A) Homology model for human β‐actin (based on PDB ID: 2BTF). Subdomains (SD) are color‐coded: SD1 (light blue), SD2 (dark blue), SD3 (dark gray) and SD4 (light gray). The nucleotide ATP, bound with magnesium, is depicted as surface representation. Key structural features are highlighted: the phosphate‐binding loops P1 (residues. 11–16; dark magenta), P2 (residues 154–161; light magenta), and the sensor loop (residues 70–78; cyan). Glycine 74 (red sphere) is located in subdomain 1 of actin and is part of the sensor loop. (B) Close‐up view of the mutation site indicated by the black square in (A), with His73, Ser74, and ATP highlighted as sticks. (C) Overview of SETD3, the actin‐specific histidine methyltransferase with bound actin peptide (PBD‐ID: 6ICV). The SET domain (light green) binds to S‐adenosyl‐methionine (SAM) and the actin sensor loop (dark green) to facilitate SAM‐dependent methyl transfer. SAM is not visible in this orientation, as it is located behind the peptide within the SET domain. The LSMT domain (light blue) likely aids in recognizing and positioning the actin protomer [50]. (D) The actin peptide, including the sensor loop, as bound to SETD3, is shown. Histidine‐73 and the surrounding residues are a hotspot for cytoskeletal actinopathy mutations causing BWCFF (marked in green and red). Histidine‐73 and tryptophan‐79 interact with binding pockets, with histidine‐73 positioned towards SAM to accept the methyl group. (E) The table lists mutations within the actin sensor loop that are known to cause BWCFF. *The sequence identity describes the proportion of 1875 analyzed eukaryotic sequences to wild‐type β‐actin. In position 76, cytoskeletal actin isoforms generally have a valine, whereas muscle isoforms have isoleucine, which results in 60% of analyzed sequences having valine in this position.

Mutations in *ACTB* and *ACTG1* disrupt the intricate network of actin function and regulation, leading to nonmuscle actinopathies, a group of rare developmental disorders [7, 8, 9]. Among these disorders, Baraitser–Winter cerebrofrontofacial syndrome (BWCFF) is attributed to missense mutations in *ACTB* or *ACTG1*, resulting in brain malformations, defects in neuronal migration, and characteristic craniofacial anomalies that include ptosis, hypertelorism, and ocular coloboma. Patients carrying *ACTB* mutations typically exhibit a more severe craniofacial phenotype compared to those with *ACTG1* variants. Beyond intellectual disabilities and developmental delays stemming from cerebral cortex malformations, BWCFF patients frequently experience muscular hypotonia, hearing loss, and a broad spectrum of additional medical issues [10, 11, 12]. The β‐actin mutation G74S, located within the sensor loop of actin (Fig. 1A,B), results in a severe BWCFF phenotype, characterized by moderate to severe intellectual disability, delayed speech, and pronounced craniofacial abnormalities [9, 11].

The sensor loop of actin (residues 70–78) is highly conserved and plays a critical role in phosphate release from filaments by sensing the nucleotide state of the protomer, thereby modulating the interaction with ABPs that differentiate based on the filament protomer's nucleotide–bound state [13, 14]. Histidine‐73 is a pivotal residue within this loop, crucial for determining the nucleotide exchange rate in monomeric actin and influencing polymerization. In yeast actin, substitution of histidine‐73 with alanine accelerates nucleotide exchange in monomeric actin and reduces the polymerization rate *in vitro*, while having no effect on the ATP hydrolysis rate [15, 16]. Histidine‐73 undergoes N3‐methylation by the actin‐specific histidine methyltransferase SET domain‐containing 3 (SETD3), which further decreases the nucleotide exchange rate and increases the polymerization rate [17, 18, 19, 20, 21]. SETD3 transfers a methyl group from S‐adenosyl‐methionine (SAM), thereby creating S‐adenosyl‐homocysteine (SAH) as a by‐product in an S_N_2 reaction [22, 23, 24]. This modification has been observed across multicellular eukaryotes; however, in yeast, it is absent [25].

SETD3 consists of a Rubisco Large Subunit Methyltransferase (LSMT) domain and a SET domain (Fig. 1C). The Rubisco LSMT domain recognizes and binds actin in a conformation that places the sensor loop residues in a position suitable for methylation in the SET domain. The SET domain accommodates both the methyl donor SAM as well as the methyl acceptor peptide, positioning histidine‐73 to accept the methyl group in proximity to SAM [20, 21, 23]. Commonly used actin purification techniques result in actin that is fully methylated at histidine 73. Consequently, most biochemical assays and structural analyses have focused on an actin‐derived peptide containing the SETD3 binding motif, leaving the methylation of native actin—especially in the context of mutations—less explored [26, 27].

In this study, we focus on the effects of the BWCFF‐causing G74S mutation in β‐actin on histidine‐73 methylation by SETD3. Molecular docking suggests that substituting glycine with serine disrupts the placement of histidine‐73 within the catalytic pocket of SETD3, implying that structural rearrangements may be necessary for binding, which could in turn hinder efficient methylation. Mass spectrometry analysis of purified recombinant human β‐actin wild‐type and G74S mutant reveals diminished histidine‐73 methylation in the mutant protein, underlining the significance of glycine‐74 in the methylation process. Furthermore, analysis of patient‐derived fibroblasts indicates that cytoskeletal wild‐type actin remains fully methylated, unaffected by the presence of mutant actin, which exhibits reduced histidine‐73 methylation itself.

## Methods

### Clinical and genomic analysis of patient data—study approval

The study was approved by the Institutional Review Board of the TU Dresden (EK‐127032017 and BO‐EK‐341062021) and local IRBs from refereeing physicians. Patients were informed of their participation prior to participating. The record of informed consent has been retained. Patients were recruited at the Institute for Clinical Genetics, University Hospital Dresden (N.DD) as part of the active patient registry within EJPRD‐funded PredACTINg project. All study methodologies involving human participants conformed to the ethical principles of the Declaration of Helsinki.

### Sequence analysis

Eukaryotic actin sequences with lengths between 370 and 380 amino acids were retrieved from the RefSeq database on July 11, 2021 [28]. Non‐actin sequences, such as actin‐binding proteins or actin‐related proteins, were excluded, resulting in a dataset of 1875 actin sequences. Multiple sequence alignment was performed using the Kalign tool provided by EMBL‐EBI [29, 30]. The region corresponding to the sensor loop in human cytoskeletal actin (residues 70–78) was analyzed in detail. The consensus sequence for this region was found to be 100% conserved across all sequences, except at position 76. At this position, 60% of the sequences contained valine, which corresponds to human cytoskeletal actin, while 40% contained isoleucine, the consensus amino acid for muscle actin isoforms. A sequence logo of the residues comprising the sensor loop was prepared using the Weblogo tool [31, 32].

### Production and purification of human SETD3 and β‐actin proteins

#### β‐actin production and purification

Human β‐actin wild‐type and the mutant β‐actin‐G74S were produced and purified as described previously [33]. For β‐actin production, a plasmid encoding human β‐actin wild‐type (UniProt ID: P60709) with a C‐terminal thymosin‐β4‐His8‐tag (UniProt ID: P62328) was used. The tag was fused to the coding sequence via an (ASR(GGS)_3_A) linker and cloned into a pFastBac‐Dual vector. The plasmid encoding β‐actin‐G74S was generated by site‐directed mutagenesis. Recombinant bacmids and baculoviruses were generated using the Bac‐to‐Bac baculovirus expression system (Thermo Fisher Scientific, Dreieich, Germany) following the manufacturer's protocol. Proteins were expressed in *Spodoptera frugiperda* (Sf9) insect cells and purified using established protocols.

#### SETD3 production and purification

Human SETD3 was produced using a plasmid encoding residues 3–502 of human SETD3 (UniProt ID: Q86TU7) with an N‐terminal His_6_‐tag in a pET28‐MHL vector (plasmid #32873; Addgene, Watertown, MA, USA). Overexpression was induced in *Escherichia coli* Rosetta2 (DE3) cells with 0.5 mm isopropyl β‐d‐1‐thiogalactopyranoside (IPTG), followed by incubation at 16 °C for 16 h. Cells were harvested and resuspended in lysis buffer (50 mm HEPES pH 7.5, 300 mm NaCl, 10 mm KCl, 1 mm DTT, 1 mm EDTA, 1 mm PMSF, 2 mm MgCl_2_, and one protease inhibitor tablet per 50 mL buffer). Lysis was performed using seven passages through a Microfluidics M‐110L microfluidizer at 40 psi. Lysates were clarified by centrifugation at 18 000 ***g*** for 30 min at 4 °C. The cleared lysate was applied to a Protino Ni‐NTA column (10 mL bed volume), washed sequentially with affinity buffer I (50 mm HEPES pH 7.5, 300 mm NaCl, 10 mm KCl, 1 mm DTT) and affinity buffer II (affinity buffer I supplemented with 60 mm imidazole). The protein was eluted with elution buffer (affinity buffer I supplemented with 150 mm imidazole). The eluted protein underwent sequential dialysis against gel filtration buffer (20 mm Na‐HEPES pH 7.2, 50 mm KCl, 1 mm DTT) at 4 °C: first in 2 L for 2 h, followed by overnight dialysis in 3 L. The His_6_‐tag was removed by treatment with TEV protease at a ratio of 1 : 100 (w/w). A final purification step was performed using size‐exclusion chromatography on a Superdex 200 Increase 17/600 column (GE Healthcare, Freiburg im Breisgau, Germany). The purified protein was concentrated to 11 mg·mL^−1^ using a Vivaspin 20 concentrator with a molecular weight cutoff of 30 kDa (Sartorius, Göttingen, Germany), flash‐frozen in liquid nitrogen with the addition of 6% sucrose as a cryoprotectant, and stored at −80 °C until use.

### Molecular docking and AI‐based structure prediction

Molecular docking experiments were performed using the crystal structure of human SETD3 bound to a human cytoskeletal actin peptide (residues 66–80) and sinefungin (PDB ID: 6OX0). The structure, representing the pre‐reactive state due to sinefungin's role as a pan‐inhibitor of S‐adenosylmethionine (SAM)‐dependent methyltransferases, was retrieved in January 2022. Sinefungin was manually replaced with the physiologically relevant cofactor SAM. To model wild‐type and mutant actin peptides, the G74S mutation was introduced into the actin peptide using coot [34]. Separate docking experiments for the wild‐type and G74S mutant actin peptides were performed using haddock 2.4 [35, 36]. Active residues for docking were selected based on their known roles in actin binding, including SETD3 residues Asn154, Arg215, Gln216, Gln255, Asp275, Thr286, Tyr288, Tyr313, and Arg316, as well as all residues of the actin peptide. Docked complexes were evaluated based on the spatial orientation and positioning of the peptide relative to its conformation in the reference crystal structure. Binding energies for the docked models were calculated using the prime mm‐gbsa v3.000 tool (Prime, Schrödinger LLC [37, 38]). Structural models of wild‐type and G74S mutant actin peptides (residues P70–M81) in complex with human methyltransferase SETD3 (UniProt ID: Q86TU7) were generated using AlphaFold 3 via the AlphaFold Server and Chai‐1 via the Chai Discovery platform (both accessed in November 2024 [39, 40]). Input sequences included both the actin peptide and the SETD3 enzyme, with default parameters applied for structure prediction. The resulting models were analyzed using Maestro (Prime, Schrödinger LLC [37, 38]) to evaluate conformational changes associated with the G74S mutation.

### Characterization of histidine methylation by SETD3

The histidine methylation activity of SETD3 was assessed using the SAMfluoro™ Methyltransferase Assay Kit (Avantor, Sigma‐Aldrich; Cat. #786‐431, Taufkirchen, Germany), following the manufacturer's instructions. This assay links the methylation reaction to the production of the fluorescent compound Resorufin, enabling real‐time monitoring of enzymatic activity.

#### Preparation of reagents

SETD3 was dialyzed overnight against reaction buffer (100 mm Tris/HCl, pH 8.0, 50 mm KCl) using a Spectrum Labs Spectra/Por 4 Dialysis Membrane (12–14 kDa cutoff). Lyophilized actin peptides (residues 66–90; Biocat, purity ≥ 95%) were dissolved in methylation assay buffer provided with the kit to a concentration of 700 μm, flash‐frozen in liquid nitrogen, and stored at −80 °C until use.

#### Reaction setup

For reactions with actin peptides, SETD3 was used at a final concentration of 3 μm and actin peptides at 30 μm. To determine Michaelis–Menten kinetics, SETD3 was used at a final concentration of 1 μm and actin peptides at concentrations between 1.25 and 100 μm. For assays with purified actin‐G74S, SETD3 and actin‐G74S were used at final concentrations of 0.1 and 10 μm, respectively. Reaction mixtures were prepared by adding 5 μL SETD3 and 20 μL substrate (actin peptides or actin‐G74S) to black flat‐bottom 96‐well plates (BrandTech Scientific, Essex, CT, USA). Reaction buffer was added to achieve a final volume of 120 μL, a final SAM concentration of 150 μm and a final ATP concentration of 20 μm.

#### Fluorescence detection

Reactions were monitored at room temperature (25 °C) using a CLARIOstar Plus microplate reader (BMG Labtech, Ortenberg, Germany). Resorufin fluorescence was measured with an excitation filter centered at 523 nm (30 nm bandwidth) and an emission filter centered at 629 nm (98 nm bandwidth), following the instrument software recommendations to optimize signal detection while minimizing background noise. For actin‐derived peptides, fluorescence was recorded using 20 flashes per cycle with a cycle time of 10 s. For native actin, faster reaction kinetics necessitated recording with 17 flashes per cycle and a cycle time of 6 s. The automatic pipetting function of the plate reader was employed for reactions involving native actin.

#### Data analysis

Reaction rates were determined from the linear portion of the fluorescence–time curve using linear regression analysis in origin 2024 software (OriginLab Corporation, Northampton, MA, USA). The rate of change in fluorescence intensity (Δfluorescence/Δ*t*) was converted to resorufin concentration using a standard curve (1.25–10 μm resorufin) generated under identical assay conditions, with measurements taken over 5 min and performed in triplicate. Fluorescence measurements were collected in triplicate for 20 min when using native actin and 60 min when using peptides, in a reaction volume of 125 μL. The amount of resorufin produced reflected the extent of histidine‐73 methylation by SETD3. Reaction rates were normalized to the molar concentration of SETD3 to calculate the enzyme's specific activity and apparent turnover number (*k*_cat_), expressed as moles of product formed per mole of enzyme per unit time.

### Protein/fibroblast MS study

Primary dermal fibroblasts from two female patients were obtained following 3 mm cutaneous punch biopsies and cultured in BIO‐AMF™‐2 Medium (Biological Industries USA, Cromwell, CT, USA). For sub‐culturing, primary fibroblasts were washed twice with 1× dPBS and detached at 37 °C for at least 3 min with 0.05% Trypsin/EDTA (Gibco^®^; Thermo Scientific, Waltham, MA, USA). Cells were resuspended in BIO‐AMF‐2 medium, seeded onto Corning plasticware (Corning, NY, USA) and maintained in BIO‐AMF‐2 medium at 37 °C in the presence of 5% CO_2_. Cultures were continued for a maximum of three passages; afterwards, the cultures were cryopreserved in multiple cryovials for long‐term storage at −150 °C. 90% FBS + 10% DMSO was used as a freezing medium. Thawing of the frozen cells was performed rapidly in a 37 °C water bath. Thawed cells were centrifuged at 240 ***g*** for 5 min and resuspended in fresh BIO‐AMF™‐2 medium into a new flask. Only cultures in early passages (maximum 7) have been used in the experiments. Cultures were labeled with the actin amino acid change and a patient ID corresponding to the ID.

#### Cell culture and lysis

Primary fibroblast cells heterozygous for the β‐actin G74S mutation (P1–P4) were cultured in BioAMF‐2 complete medium (NeoFroxx) using 25 cm^2^ flasks (Corning) as described earlier [8]. At ~ 50% confluency, cells were washed twice with phosphate‐buffered saline (PBS), detached with 0.05% Trypsin/EDTA (Thermo Scientific), and centrifuged (5 min, 500 ***g***). Pellets were resuspended in ice‐cold lysis buffer (50 mm Tris/HCl pH 7.5, 150 mm NaCl, 1 mm EDTA, 1% NP‐40, 10% glycerol, protease inhibitor cocktail) at 8000 cells·μL^−1^. Lysates were sonicated (10 min), homogenized by pipetting, aliquoted, and stored at −20 °C. Protein integrity was verified by SDS/PAGE prior to mass spectrometry.

#### In‐gel digestion

Actin bands were excised from Coomassie‐stained polyacrylamide gels, subjected to in‐gel tryptic digestion, and peptides were extracted for analysis.

#### Mass spectrometric analysis and data processing

RP‐LC–MS/MS was performed essentially as described previously (https://doi.org/10.1002/pmic.202400239). Briefly, a nano‐flow UltiMate 3000 RSLCnano LC system (Thermo Fisher Scientific) equipped with a trapping column (3 μm C18 particle, 2 cm length, 75 μm ID, Acclaim PepMap) and a 50 cm μPAC™ analytical column (Thermo Fisher Scientific) was used and coupled online to an Orbitrap Exploris™ 240 mass spectrometer. Solvent A consisted of 0.1% formic acid in water, and solvent B of 80% acetonitrile with 0.1% formic acid. Samples were injected onto the trapping column at a flow rate of 15 μL·min^−1^ using 2.5% solvent B for 3 min for enrichment and desalting. The trapping column was then switched online with the analytical column, and peptides were eluted using the following multi‐step linear gradient of solvent B at a flow rate of 0.5 μL·min^−1^ and a column temperature of 35 °C: from 2.5% to 25% in 60 min, from 25% to 50% in 32 min, from 50% to 95% in 5 min, held at 95% for 4 min, decreased to 2.5% in 1 min, and re‐equilibrated at 2.5% for an additional 15 min. Full MS scans were acquired at a resolution of 60 000 over a mass‐to‐charge ratio (*m/z*) range of 300–1900. Precursor ions were excluded from repeated fragmentation using dynamic exclusion (70 s duration, 10 p.p.m. mass tolerance window). MS/MS spectra were acquired with a 1.6 *m/z* isolation window, a resolution of 15k, and a maximum injection time of 100 ms. Raw data were processed using freestyle™ software (version 1.8 SP2 QF1; Thermo Fisher Scientific). Extracted ion chromatograms (EICs) were generated by querying calculated *m/z* values of doubly charged modified tryptic peptides against chromatographic profiles, applying a ±5 p.p.m. mass tolerance. EIC peaks were validated by manual inspection of MS and MS/MS spectra.

#### Data analysis

Data analysis and graph plotting were performed with origin 2024 (OriginLab Corporation). Errors are given as standard deviation (SD) based on three independent experiments if not otherwise specified. The significance of the data was evaluated in origin 2024 using a two‐sample *t*‐test (*P* > 0.05 ≙ ns, *P* ≤ 0.05 ≙ *, *P* ≤ 0.01 ≙ **, *P* ≤ 0.001 ≙ ***).

## Results

### The actin sensor loop as a hotspot for BWCFF‐associated mutations

Comparative analysis of 1875 eukaryotic actin sequences revealed conservation of mutation relevant residues of the sensor loop, with a single exception at position 76, where valine is substituted by isoleucine in muscle‐specific isoforms. This remarkable evolutionary conservation highlights the critical functional importance of this structural element (Fig. S1). While mutations associated with BWCFF are dispersed throughout the sequence and structure of cytoskeletal β‐ and γ‐actin isoforms [41], a distinct cluster of nine mutations affecting His73 and adjacent residues within the sensor loop (residues 70–78) has been exclusively linked to BWCFF or microlissencephaly (Fig. 1D,E) [9, 11, 42, 43, 44]. Intriguingly, mutations in the homologous region of other actin isoforms, such as skeletal α‐actin (ACTA1), are frequently associated with severe forms of nemaline myopathy, a congenital myopathy characterized by muscle weakness and the presence of nemaline bodies in muscle fibers [45, 46, 47].

### The G74S mutation impairs histidine‐73 binding to SETD3

To assess whether the G74S mutation affects the binding of histidine‐73 to the actin‐specific methyltransferase SETD3, we employed structural modeling and molecular docking approaches. Using a previously reported SETD3 structure complexed with an actin‐derived peptide [48, 49], we substituted the pan‐inhibitor sinefungin with the physiologically relevant S‐adenosyl‐methionine (SAM) and introduced the G74S mutation.

**Fig. 2:**
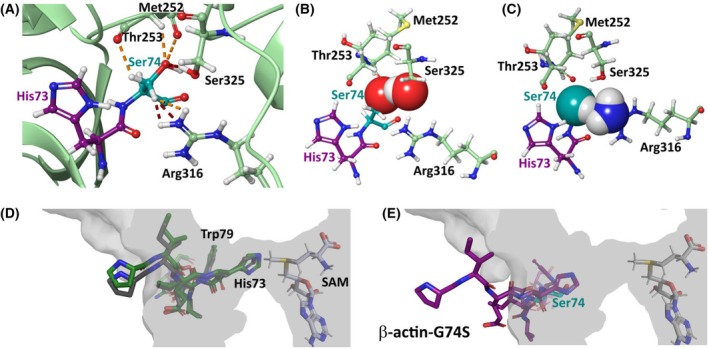
The actin mutation to serine in position 74 hinders binding of histidine‐73 to SETD3. (A–C) The mutation to serine (cyan) in the actin peptide (magenta) results in steric clashes with SETD3 (light green), which impairs identical binding of histidine‐73 to the methyltransferase as for the wild‐type peptide. Residue numbers refer to the human SETD3 sequence (UniProt ID: Q86TU7). (A) Overview of the binding site for histidine‐73 (magenta) and serine‐74 (cyan) in complex with SETD3. The mutation introduces steric conflicts between the side chain of actin‐serine‐74 and atoms from SETD3. Notably, there are substantial vdW clashes of arginine‐316 and serine‐325 (red dotted lines), as well as other smaller clashes with methionine‐252, threonine‐253, and arginine‐316 (orange dotted lines). (B) Close‐up of the vdW overlap between the hydroxyl group of serine‐74 and the hydroxyl group of serine‐325, showing the vdW radii of these atoms. (C) Close‐up of the vdW overlaps between serine‐74 and arginine‐316, with the vdW radii of the hydroxyl group of serine‐74 and the amino group of arginine‐316 shown. (D, E) Molecular docking of an actin peptide to SETD3 (PDB ID: 6ICV) was performed using Haddock2.4. A cross‐section of SETD3 (gray) shows the histidine‐73 binding groove and SAM (gray sticks). (D) Docking of the wild‐type actin peptide (dark gray) results in a conformation comparable to the crystal structure (dark green), with histidine‐73 and tryptophan‐79 binding into their respective binding pockets. The Nδ1 atom of histidine‐73 is positioned to accept the methyl group still bound to SAM. In the docked wild‐type structure, the distance between Nδ1 and the methyl carbonyl atom is 2.6 Å, closely matching the 2.1 Å observed in the crystal structure. (E) Docking of the mutated actin peptide (magenta) shows that serine‐74 (cyan) disrupts peptide binding. Although the peptide maintains a similar overall conformation, neither histidine‐73 nor tryptophan‐79 are correctly positioned within their binding pockets. In the mutated complex, the Nδ1 to methyl carbonyl distance increases significantly to 7.9 Å.

Structural modeling revealed that the G74S substitution creates steric clashes within the SETD3 binding site (Fig. 2A). Specifically, van der Waals (vdW) overlap analysis showed severe clashes between the hydroxyl group of serine‐74 and the hydroxyl group of SETD3 serine‐325 (*d* = 1.72 Å, *o* = 0.37; Fig. 2B) as well as between the methylene group of serine‐74 and the amino group of SETD3 arginine‐316 (*d* = 1.55 Å, *o* = 0.35; Fig. 2C). Additional vdW overlaps further hindered proper peptide positioning. Even after energy minimization, steric hindrances persisted, indicating that the mutant peptide cannot adopt the correct binding conformation.

### Molecular docking and AI‐based structure prediction confirm impaired binding of the mutant peptide

To assess whether the mutant peptide compensates for steric hindrances through conformational adjustments, molecular docking analyses were conducted. Docking of the wild‐type peptide recapitulated its experimentally determined binding conformation, with histidine‐73 and tryptophan‐79 correctly positioned within their respective binding pockets. The calculated binding energy for the wild‐type complex was −125.98 kcal·mol^−1^, indicating strong interaction (Fig. 2D). In contrast, docking of the G74S mutant peptide led to a misalignment of histidine‐73 and tryptophan‐79, leading to a substantial decrease in binding energy (−82.04 kcal·mol^−1^; Fig. 2E).

**Fig. 3:**
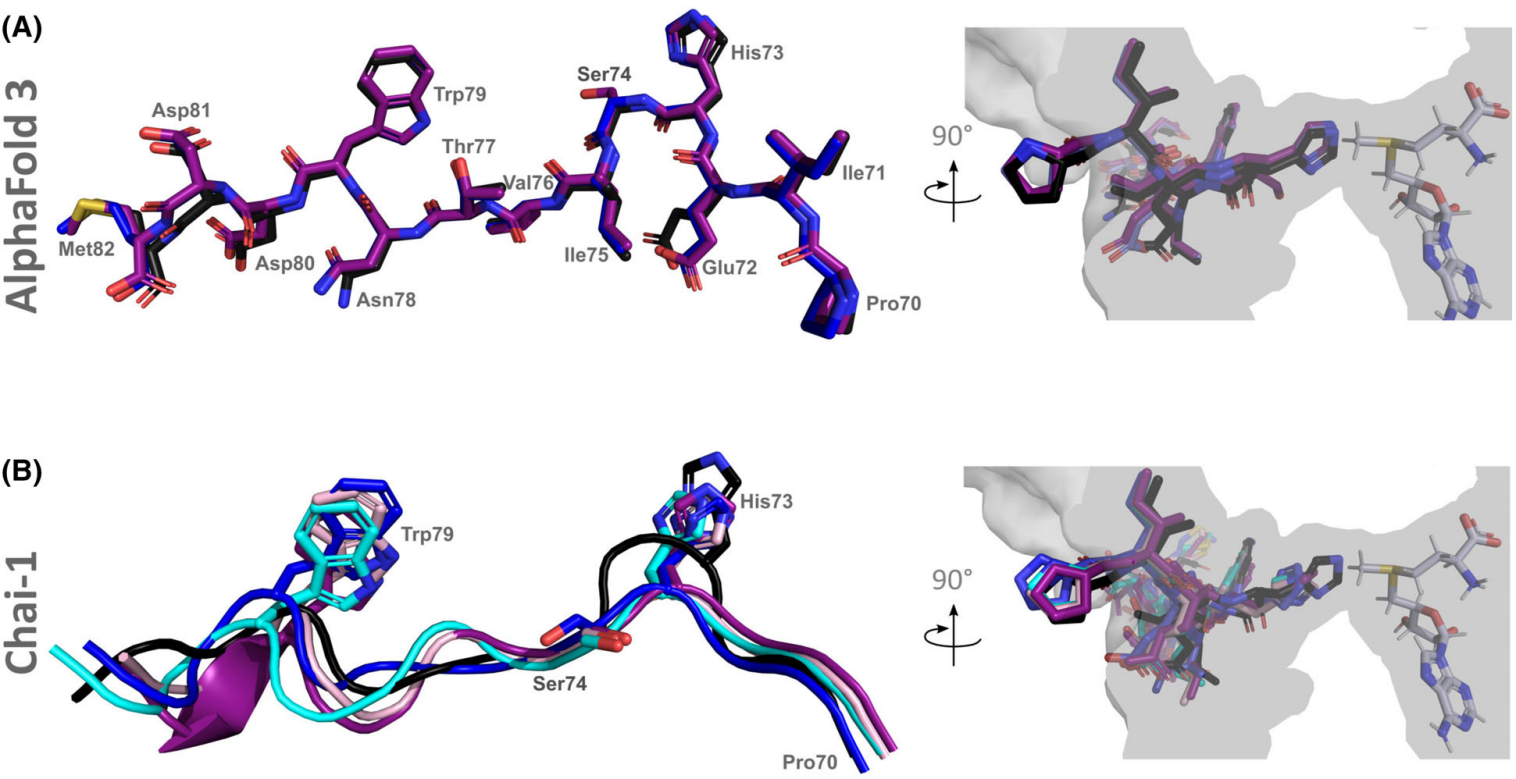
AI‐based structure comparison. (A) Comparison of SETD3 crystal structure bound to the actin‐wt peptide with AF3 prediction of SETD3 bound to the actin‐G74S peptide. The peptide from the crystal structure is depicted in black, from the structure predictions colored, SETD3 residues from the crystal structure are depicted in light green, from the structure prediction in light blue. Structural alignment of the peptide from the crystal structure with the ones from the structure prediction. (B) Comparison of SETD3 crystal structure bound to the actin‐wt peptide with Chai‐1 prediction of SETD3 bound to the actin‐G74S peptide. The peptide from the crystal structure is depicted in black, from the structure prediction colored, SETD3 residues from the crystal structure are depicted in light green, from the structure prediction in light blue. Structural alignment of the peptide from the crystal structure with the ones from the structure prediction.

These findings suggest that the G74S mutation disrupts SETD3 binding, thereby impairing the methylation reaction. To further explore potential structural consequences, we employed AI‐based structure predictions using AlphaFold 3 (AF3) and Chai‐1 [39, 40]. AF3 models indicate that, with minor rearrangements in SETD3, the mutant peptide may adopt a conformation resembling the wild‐type complex (Fig. 3A, Fig. S2A–C), thus remaining compatible with methylation. In contrast, Chai‐1 models depict the mutant peptide in alternative poses, where histidine‐73 approaches—but does not fully engage—its binding site (Fig. 3B, Fig. S2D–F). These predictions suggest that the binding of the mutant peptide may require structural adaptation in SETD3, while binding without vdW overlaps is possible, consistent with the reduced methylation efficiency observed *in vitro*.

### The G74S mutation reduces SETD3 methylation efficiency

**Fig. 4:**
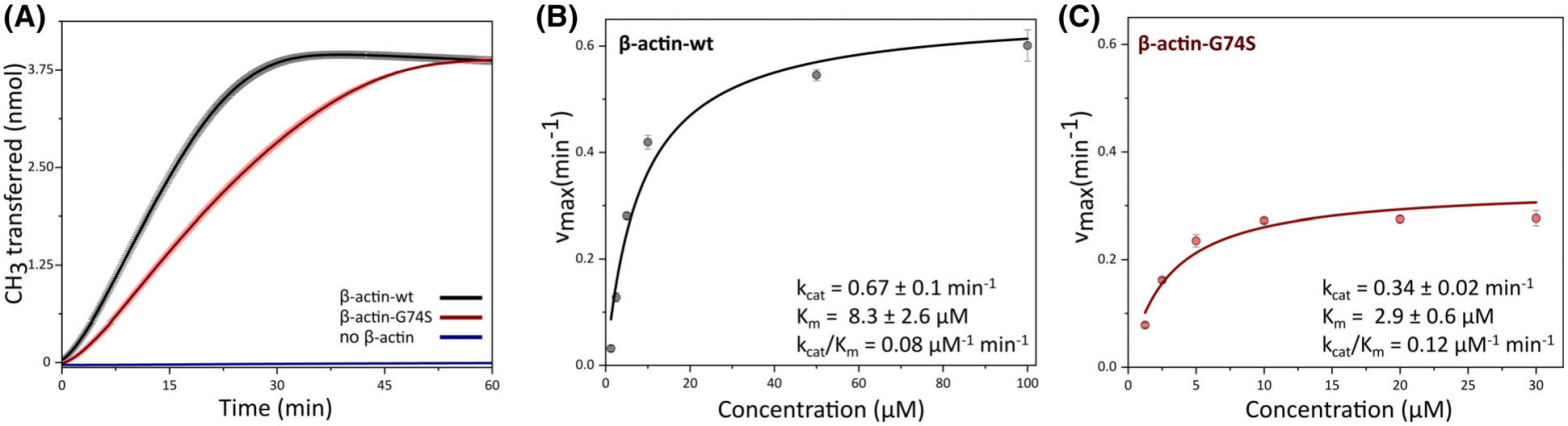
SETD3 activity is reduced in the presence of the G74S mutation in actin peptides. (A) Methylation of histidine‐73 within 30 μm of a β‐actin‐derived peptide (residues 66–80), containing the SETD3 binding motif, was monitored using an enzyme‐coupled assay. The assay detects the conversion of the reaction byproduct S‐adenosylhomocysteine (SAH) into the fluorophore resorufin through several catalytic steps. Initial reaction rates were determined from the linear portion of the fluorescence–time curve by calculating the change in fluorescence intensity over time (∆fluorescence/∆*t*). Fluorescence values were converted to resorufin concentrations using a standard curve (1.25–10 μm resorufin) generated under identical assay conditions, with measurements performed in triplicate over 5 min. The amount of resorufin produced was used to quantify histidine‐73 methylation catalyzed by SETD3. Reaction rates were normalized to the molar concentration of SETD3 to calculate the turnover number, expressed as the number of substrate molecules converted per enzyme molecule per time unit. The observed turnover number *k*_cat_ was 0.43 ± 0.01 min^−1^ for the wild‐type peptide and 0.25 ± 0.01 min^−1^ for the G74S mutant peptide, indicating a significant reduction in SETD3 catalytic activity due to the G74S substitution. (B) Enzyme kinetics of SETD3 with actin‐derived wild‐type peptide. Initial velocities were measured over a range of substrate concentrations; data were fitted to the Michaelis–Menten equation to determine *K*_m_ and *k*_cat_ values. Each data point represents the mean ± SD of three independent replicates. (C) Enzyme kinetics of SETD3 with actin‐derived G74S mutant peptide. Initial velocities were measured over a range of substrate concentrations; data were fitted to the Michaelis–Menten equation to determine *K*_m_ and *k*_cat_ values. Each data point represents the mean ± SD of three independent replicates.

To quantify the effect of the G74S mutation on SETD3 enzymatic activity, we performed a SAM‐dependent methylation assay using actin‐derived peptides (residues 66–80). Methylation efficiency was assessed using an enzyme‐coupled fluorescence assay that detects S‐adenosyl‐homocysteine (SAH) formation. The observed turnover number *k*_cat_ for SETD3 methylation of histidine‐73 in the wild‐type peptide was 0.67 ± 0.1 min^−1^, consistent with previously reported values [21, 22]. In contrast, the measured *K*_m_ of 8.3 ± 2.6 μm was approximately 3‐fold lower than the earlier reported value of 24 ± 2 μm. These modest discrepancies likely reflect differences in assay conditions, enzyme source, or purification protocols, all of which are known to affect kinetic parameters [27]. The G74S mutant peptide exhibited a 49.6% reduction in *k*_cat_ (0.34 ± 0.02 min^−1^), indicating that the mutation significantly impairs the methylation efficiency (Fig. 4).

### Mass spectrometry confirms decreased histidine‐73 methylation in recombinant and patient‐derived β‐actin

**Fig. 5:**
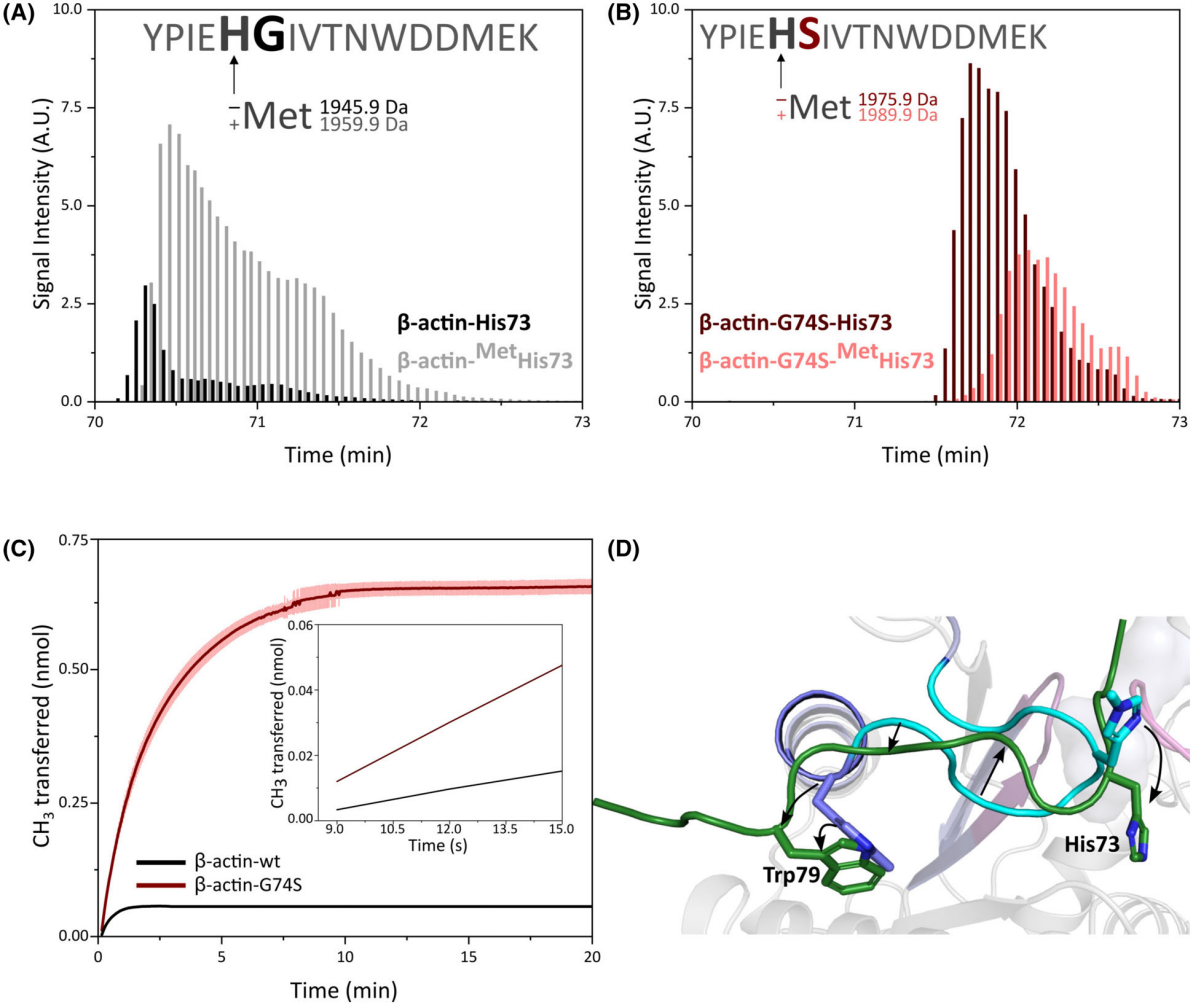
Actin mutation G74S impairs histidine‐73 methylation in recombinantly produced proteins. (A) Mass spectrometry analysis of wild‐type β‐actin (β‐actin‐WT) produced in the *Sf9* baculovirus expression system. The actin peptide methylated at histidine‐73 is shown in gray, while the non‐methylated form is shown in black. Approximately 89% of β‐actin‐WT is methylated at histidine‐73. (B) Mass spectrometry analysis of β‐actin‐G74S, also expressed in Sf9 cells. The peptide methylated at histidine‐73 is depicted in light red, and the non‐methylated peptide in dark red. Approximately 32% of β‐actin‐G74S is methylated at histidine‐73. (C) Methylation of histidine‐73 in 10 μm recombinant β‐actin‐G74S by SETD3 was monitored using an enzyme‐coupled assay, based on the conversion of SAH to resorufin (as described in Fig. 2). The average signal (dark red line) and standard deviation (light red lines) from three technical replicates are shown for recombinant β‐actin‐G74S, and the signal of two technical replicates for β‐actin‐WT is shown in black. The observed turnover number *k*_cat_ was 16 ± 1 min^−1^ for the wild‐type protein and 26 ± 1 min^−1^ for the G74S mutant protein. The turnover number for the wild‐type protein is lower compared to the mutant protein, as the concentration of non‐methylated histidine‐73 is considerably lower, and about 90% pre‐methylated protein leads to product inhibition. (D) A structural alignment is shown between the actin peptide bound to SETD3 (dark green) and the corresponding region within the fully folded actin monomer. The remainder of the actin structure, which spatially surrounds the peptide region, is rendered as a transparent cartoon to provide structural context. The P1‐loop, P2‐loop, and sensor loop are highlighted in dark magenta, light magenta, and cyan, respectively. Residues corresponding to the actin peptide in the SETD3‐bound conformation are highlighted in light blue, consistent with the color scheme used in Fig. 1. The side chains of histidine‐73 and tryptophan‐79 are depicted as stick models, and black arrows denote the positional shifts undergone by actin residues upon SETD3 binding.

To confirm the impact of the G74S mutation on histidine‐73 methylation, we quantitatively analyzed methylation levels in recombinantly expressed wild‐type and G74S β‐actin using mass spectrometry (MS). Recombinant β‐actin was purified from *S. frugiperda* (*Sf9*) cells, subjected to SDS/PAGE, digested with trypsin, and analyzed by reversed‐phase liquid chromatography–tandem mass spectrometry (RP‐LC–MS/MS). MS analysis revealed that 84% of the wild‐type β‐actin target peptide (residues Tyr69‐Lys84) was methylated at histidine‐73 (Fig. 5A), whereas only 33% of the corresponding peptide from G74S mutant β‐actin was methylated (Fig. 5B). These findings demonstrate that SETD3‐mediated histidine‐73 methylation is significantly reduced in the G74S mutant.

To determine whether the remaining 67% of unmethylated G74S mutant β‐actin and 16% of wild‐type β‐actin could still be methylated post‐production—or whether structural changes during folding prevented methylation—we conducted methylation assays using actin purified from *Sf9* cells (Fig. 5C). Both wild‐type and G74S mutant β‐actin were methylated by SETD3, but to different extents, corresponding to the proportions of previously unmethylated actin in the samples (observed turnover number *k*_cat_: wild‐type β‐actin, 16 ± 1 min^−1^; G74S mutant β‐actin, 26 ± 1 min^−1^). Structural comparison between the SETD3‐bound actin peptide and the folded actin monomer indicates that only limited conformational adjustments may be needed to allow methylation. In particular, modeling suggests side chain flipping of histidine‐73 and tryptophan‐79, along with repositioning of the intervening loop, to accommodate binding within the catalytic pocket of SETD3 (Fig. 5D).

**Fig. 6:**
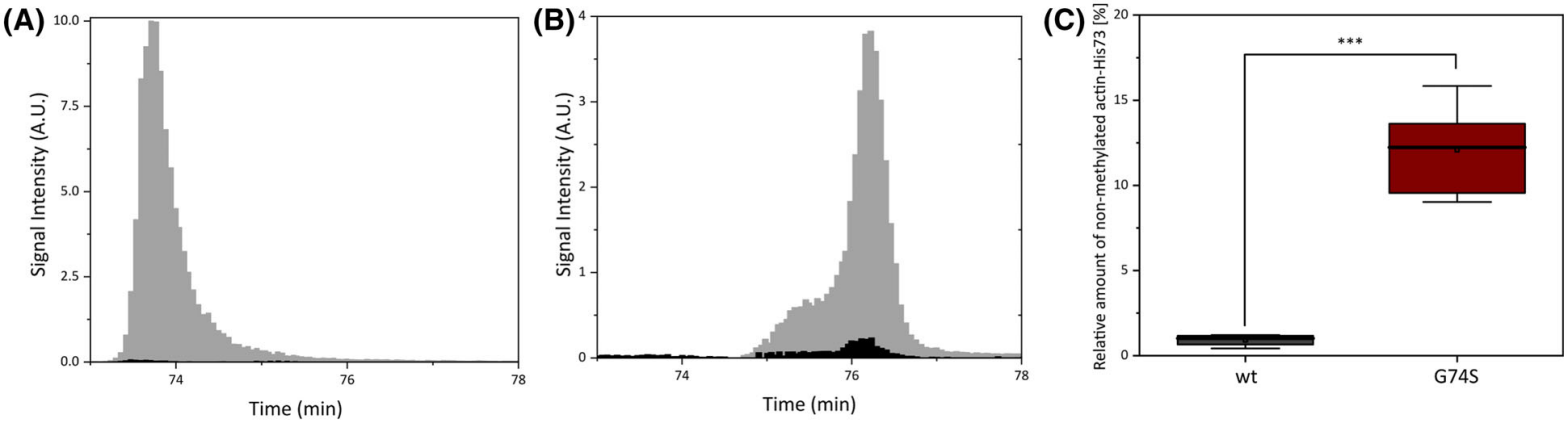
Histidine‐73 methylation in patient‐derived fibroblasts with the heterozygous β‐actin‐G74S mutation. Fibroblast lysates from a patient with the heterozygous β‐actin‐G74S mutation were analyzed for histidine‐73 methylation using mass spectrometry. The proteins were separated by SDS/PAGE and the actin band subjected to trypsin digestion; peptides comprising actin residues 69–84 were identified using mass spectrometry analysis. The peak signal intensity for peptides containing methylated (black) and non‐methylated (gray) histidine‐73 is shown for (A) β‐actin‐wt and (B) β‐actin‐G74S peptides. (C) Box plot showing the proportion of six independent measurements. Data include both oxidized and non‐oxidized forms of the peptides. Despite being produced in the same cells, β‐actin‐G74S shows markedly lower levels of histidine‐73 methylation compared to β‐actin‐wt, consistent with impaired SETD3‐mediated methylation due to steric hindrances caused by the G74S mutation (two‐sample *t*‐test *P* ≤ 0.001 ≙ ***).

To examine whether this effect extends to endogenously expressed β‐actin, we analyzed patient‐derived fibroblasts by MS. In control fibroblasts, the target peptide was almost fully methylated at histidine‐73, with only ~ 1% of the total actin pool lacking this modification (Fig. 6A,C). However, in BWCFF patient‐derived fibroblasts expressing the G74S β‐actin mutant, 12% of the actin pool was non‐methylated (Fig. 6B,C).

Collectively, our results demonstrate that the G74S mutation in β‐actin negatively affects histidine‐73 methylation, both in recombinant and endogenous contexts. While the reduction in methylation is more pronounced in purified recombinant protein, the mutation still significantly impairs histidine‐73 methylation in patient‐derived fibroblasts. These findings provide mechanistic insight into how the G74S mutation may impair SETD3‐mediated posttranslational modification, potentially contributing to the molecular pathology of BWCFF.

## Discussion

The aim of our study was to investigate the effect of the BWCFF‐causing β‐actin mutation G74S on histidine‐73 methylation by the methyltransferase SETD3. Our results confirm previous observations that changes within the SETD3 binding motif of actin are critical for proper substrate recognition and efficient histidine‐73 methylation [22, 23, 24, 26, 27]. Our data indicate that the G74S mutation likely disrupts proper peptide binding to the SETD3 catalytic domain by introducing steric hindrances, which in turn reduces the efficiency of methylation. Although conformational adjustments in the SETD3 active site can partially circumvent these steric hindrances, the overall efficiency of histidine‐73 methylation is compromised, resulting in reduced levels of methylated recombinant mutant actin. Notably, our results suggest that the G74S actin mutation generates two aberrant species in cells: one that is histidine‐73 methylated and one that is not. To our knowledge, this is the first reported cytoskeletal actinopathy mutation that directly affects a posttranslational modification, highlighting the need to consider such modifications when evaluating actinopathy‐related mutations. Importantly, our data also show that SETD3 can directly methylate native mutant actin in the presence of SAM, without the need for additional cofactors.

The reduced methylation observed for G74S actin in insect cells is consistent with a slower catalytic rate due to the structural rearrangements required for binding and positioning within the SETD3 active site. While 84% of wild‐type actin was methylated, only 33% of the G74S variant underwent modification during overexpression, likely reflecting a kinetic limitation during folding and assembly rather than a loss of substrate compatibility. This is further supported by *in vitro* methylation of the purified mutant protein, which confirms that SETD3 can still methylate the G74S variant efficiently when provided sufficient time and enzyme access. These results indicate that the G74S mutation does not prevent methylation, but rather slows the reaction sufficiently to reduce modification levels in the cellular context of overexpression.

While our study provides important insights into the mechanistic effects of the G74S mutation, several limitations should be acknowledged. The majority of our analyses were performed using recombinant β‐actin and cultured fibroblasts. Recombinant proteins do not fully recapitulate the cellular context, and fibroblast cultures may not reflect tissue‐specific differences in actin turnover, methylation dynamics, or the modulatory effects of actin‐binding proteins. Future studies using animal models or primary patient cells will be essential to gain more physiologically relevant insights.

Moreover, although we observed a reduction in histidine methylation for the G74S mutant in cellular contexts, the functional consequences of this reduction remain speculative. We hypothesize that reduced methylation may affect actin polymerization or actin‐binding protein interactions, but further investigation—such as detailed analyses of cytoskeletal dynamics, cell migration, and actin polymerization in cells expressing the G74S mutation—is required to confirm these effects.

Another aspect that warrants further investigation is the potential compensatory mechanisms that cells might employ to mitigate the effects of the G74S mutation. Identifying and characterizing these compensatory factors could not only deepen our understanding of actin regulation, but also reveal novel therapeutic targets.

The multiple effects of the G74S mutation are likely to go beyond a reduction in methylation rate. In tissues with high actin turnover, such as migrating fibroblasts or rapidly proliferating cancer cells, the fraction of unmethylated β‐actin G74S may be higher, contributing to a heterogeneous actin pool composed of histidine‐73‐methylated wild‐type actin, histidine‐73‐methylated G74S actin, and unmethylated G74S actin. This heterogeneity could influence cytoskeletal dynamics and cellular function over time, even if the observed reduction in methylation is relatively modest. Given that histidine methylation has been implicated in modulating nucleotide exchange, polymerization rates, and interactions with actin‐binding proteins [15, 16], even small deficits in this post‐translational modification could have cumulative effects on cytoskeletal organization leading to disease [21].

In conclusion, our results establish a direct link between a cytoskeletal actinopathy mutation and altered posttranslational modification, highlighting the importance of considering histidine methylation in the context of β‐actin‐related diseases. Future research should focus on dissecting the interplay between methylation status and actin dynamics, as well as exploring therapeutic strategies that might enhance SETD3 activity or mimic histidine methylation to counteract the deleterious effects of the G74S mutation.

## Author contributions

AM, FFRB, and DJM designed the methodology. AM, FFRB, and DJM contributed to validation and formal analysis. AM and FFRB carried out experimental investigation. AM, MSM, and PMP contributed to the cell biology experiments, while AM, JNG, and MHT conducted biochemical analyses. AM performed the *in silico* studies and managed data curation. AM and JK analyzed *in silico* data. AM and DJM drafted the original manuscript and prepared visualizations. NDD conducted the clinical evaluations. NDD and DJM secured funding. DJM oversaw conceptualization, resource provision, supervision, and project administration. All authors reviewed and approved the final manuscript.

## Peer review

The peer review history for this article is available at https://www.webofscience.com/api/gateway/wos/peer‐review/10.1002/1873‐3468.70088.

## Supporting information


**Fig. S1.** Sequence logo of the actin sensor loop.
**Fig. S2.** AI‐bases structure comparison.

## Data Availability

The data that supports the findings of this study are available in Figs 1, 2, 3, 4, 5, 6 and the Supporting Information of this article. Additional data, which are not publicly available due to privacy or ethical restrictions, can be obtained upon request from the corresponding author.
