# Supplementary material for: Disruption of SETD3‐mediated histidine‐73 methylation by the BWCFF‐associated β‐actin G74S mutation
Source: FEBS Lett. 2025 Jun 9;599(17):2449–62. doi: 10.1002/1873-3468.70088 (PMC12421705; doi:10.1002/1873-3468.70088)
Supplement: Supplementary file 1 — Fig. S1. Sequence logo of the actin sensor loop. Fig. S2. AI‐bases structure comparison. [file FEB2-599-2449-s001.pdf]

Supplementary Figures

"Disruption of SETD3-mediated histidine-73 methylation by the BWCFF-associated β-actin G74S mutation"

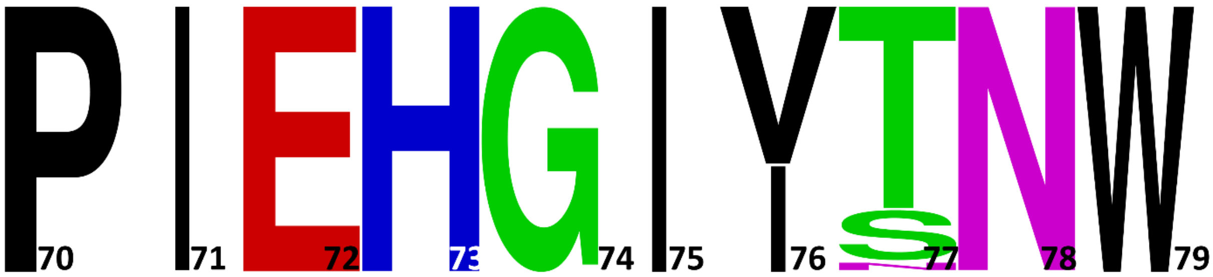

Supplementary Figure 1: Sequence logo of the actin sensor loop. Conservation and variability of amino acids of the actin sensor loop (P70 – W79) are shown. Positions that are known for BWCFF mutation sites have 100% conservation in their respective position.

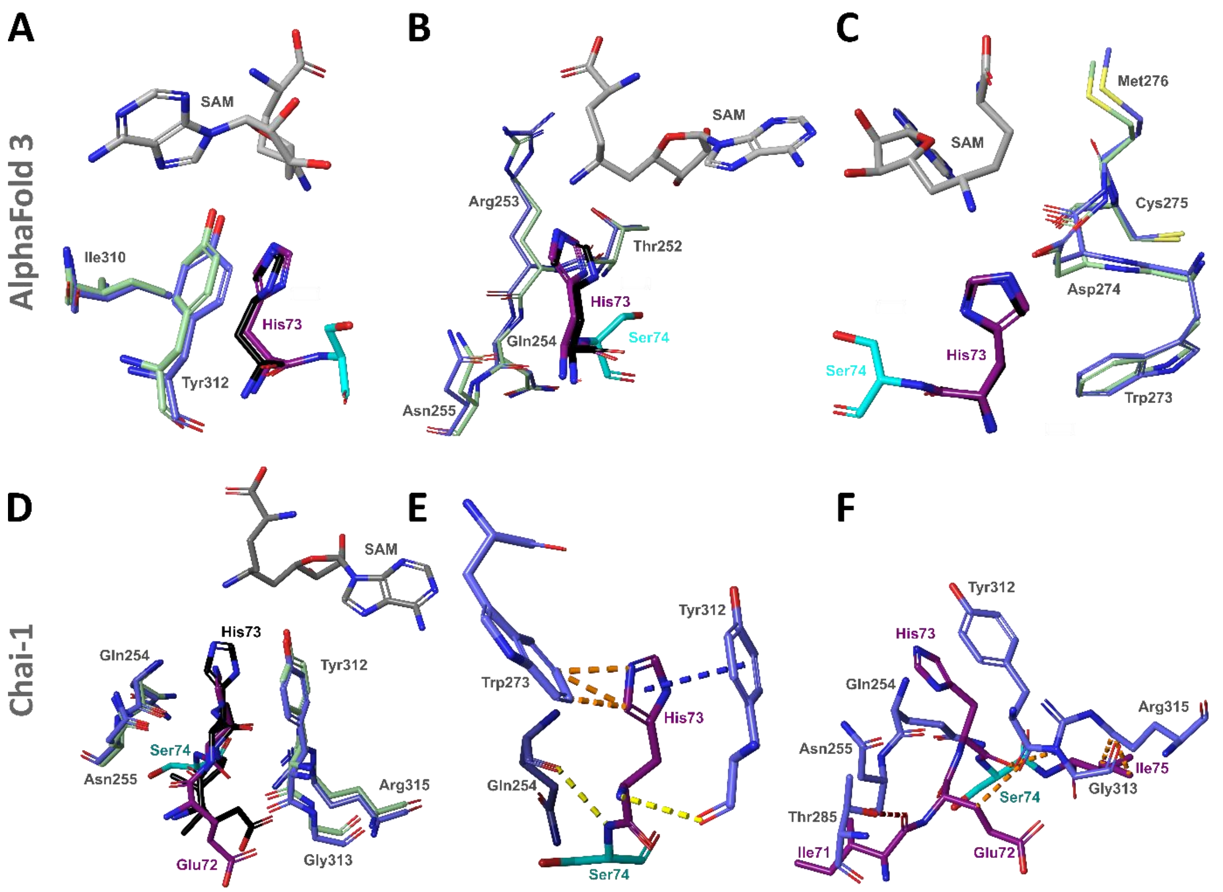

Supplementary Figure 2: AI-bases structure comparison. (A-C) Comparison of predicted residue positionings of AF3 models of SETD3 bound to the actin-G74S peptide to the crystal structure of SETD3 bound to the actin-wt peptide. The peptide from the crystal structure is depicted in black, from the structure predictions colored, SETD3 residues from the crystal structure are depicted in light green, from the structure prediction in light blue. **(D-F)** Comparison of SETD3 crystal structure bound to the actin-wt peptide with Chai-1 prediction of SETD3 bound to the actin-G74S peptide. The peptide from the crystal structure is depicted in black, from the structure prediction colored, SETD3 residues from the crystal structure are depicted in light green, from the structure prediction in light blue. **(D)** Comparison of predicted residue positions of one Chai-1 predicted model to the crystal structure. **(E-F)** Positioning of residues of one Chai-1 predicted model shows several vdW overlaps, which can be resolved by energy minimization.
